# Genome Sequence of Rhodococcus erythropolis Type Strain JCM 3201

**DOI:** 10.1128/MRA.01730-18

**Published:** 2019-04-04

**Authors:** Keitaro Yoshida, Wataru Kitagawa, Koji Ishiya, Yasuo Mitani, Nobutaka Nakashima, Sachiyo Aburatani, Tomohiro Tamura

**Affiliations:** a Bioproduction Research Institute, National Institute of Advanced Industrial Science and Technology (AIST), Sapporo, Japan; b Computational Bio Big-Data Open Innovation Laboratory (CBBD-OIL), AIST, Tokyo, Japan; c Graduate School of Agriculture, Hokkaido University, Sapporo, Japan; d Biotechnology Research Institute for Drug Discovery, AIST, Tokyo, Japan; University of California, Riverside

## Abstract

Rhodococcus erythropolis JCM 3201 can express several recombinant proteins that are difficult to express in Escherichia coli. It is used as one of the hosts for protein expression and bioconversion. Here, we report the draft genome sequence of R. erythropolis JCM 3201.

## Abstract (precis)

Rhodococcus erythropolis JCM 3201 can express several recombinant proteins that are difficult to express in Escherichia coli. It is used as one of the hosts for protein expression and bioconversion.

## ANNOUNCEMENT

Rhodococci remarkably degrade various xenobiotics (1–3). The type strain of Rhodococcus erythropolis, JCM 3201, was originally isolated from soil as a degrader of aromatics (4). A lysozyme-sensitive mutant of JCM 3201, strain L88, has been used as an expression host of recombinant proteins that are difficult to express in Escherichia coli (5–9). JCM 3201 is also useful to the host for bioconversion of chemicals such as vitamin D_3_ to hydroxyvitamin D_3_ (10–12). To optimize these processes thorough metabolic pathway engineering, its genomic information will be essential. The draft genome of JCM 3201 was previously deposited with 67 contigs (GenBank accession number BCRM00000000). However, this sequence lacks information about the numbers and structural type of its chromosomes and plasmids.

To improve the genome assembly level, we resequenced the JCM 3201 genome. We cultured the strain in LB broth containing 1% glycine, lysed cells with 2 mg ml^−1^ lysozyme, and extracted the genomic DNA by the phenol-chloroform method (13). We prepared three libraries with the TruSeq DNA PCR-free library prep kit (Illumina), the Nextera mate pair sample preparation kit (Illumina), and the DNA template prep kit version 1.0 (PacBio). The genome was sequenced using (i) an Illumina HiSeq 2500 platform with 350-bp paired-end and 8-kbp mate pair libraries and (ii) a PacBio RS II platform. The PacBio raw reads were filtered with SMRT Analysis version 2.3.0 with the following parameters: minimum subread length, 500; minimum polymerase read quality, 0.80; and minimum polymerase read length, 100. We obtained 98,981 subreads and assembled them with the Hierarchical Genome Assembly Process (HGAP) version 2 (14), resulting in a 6.33-Mbp circular sequence with a mean coverage of 84-fold. The paired-end and mate pair reads were filtered with Trimmomatic version 0.38 with the parameters SLIDINGWINDOW:20:20 and MINLEN:50 (15), resulting in 14,661,238 and 9,431,622 reads, respectively. We assembled these reads using Velvet version 1.2.08 (16) and obtained 52 contigs. Among these contigs, 12 did not match the circular PacBio assembly, which showed the highest similarities to plasmid sequences of other R. erythropolis strains (17). To confirm the possibility that these contigs were derived from plasmid sequences, we examined their connectivity by PCR and finally obtained another 85-kb circular sequence and a 241-kb linear sequence. Based on their sizes, we predicted that these two sequences were plasmids. One end of the linear sequence showed similarity to end sequences of linear plasmids in rhodococci. However, the other end did not, suggesting that this linear sequence might not be completed yet. To polish the assembled sequences, we mapped the filtered paired-end reads to the initial assembly using the Burrows-Wheeler Aligner MEM algorithm (BWA-MEM) version 0.7.12 with a seed length of 19 nucleotides (18) and corrected errors using the Genome Analysis Toolkit (GATK) version 4.0.6.0 of variant filtration with default parameters (19). We annotated the genes using DFAST (20).

The draft genome sequence of JCM 3201 contained two circular sequences and a linear sequence with a length of 6,326,569 bp (62.4% G+C content), 84,587 bp (62.5% G+C content), and 240,958 bp (61.2% G+C content), respectively. It contains 6,152 putative coding DNA sequences (CDSs), 12 rRNAs, and 53 tRNAs.

### Data availability.

The DDBJ/EMBL/GenBank sequence accession numbers for this project are BHXB01000001 to BHXB01000003. The SRA accession numbers are DRX143934 to DRX143936.
